# Outcomes Among African American and Non-Hispanic White Men With Metastatic Castration-Resistant Prostate Cancer With First-Line Abiraterone

**DOI:** 10.1001/jamanetworkopen.2021.42093

**Published:** 2022-01-05

**Authors:** Mallika Marar, Qi Long, Ronac Mamtani, Vivek Narayan, Neha Vapiwala, Ravi B. Parikh

**Affiliations:** 1 Department of Radiation Oncology, Stanford University, Stanford, California; 2 Perelman School of Medicine, Department of Radiation Oncology, University of Pennsylvania, Philadelphia; 3 Perelman School of Medicine, Department of Biostatistics, Epidemiology and Informatics, University of Pennsylvania, Philadelphia; 4 Perelman School of Medicine, Department of Medicine, University of Pennsylvania, Philadelphia; 5 Perelman School of Medicine, Department of Medical Ethics and Health Policy, University of Pennsylvania, Philadelphia; 6 Corporal Michael J. Crescenz VA Medical Center, Philadelphia, Pennsylvania

## Abstract

**Importance:**

Prospective evidence suggests abiraterone is associated with superior progression-free survival for African American men compared with non-Hispanic White men with metastatic castration-resistant prostate cancer (mCRPC).

**Objective:**

To investigate differences in outcomes with first-line abiraterone therapy between African American and non-Hispanic White men with mCRPC in a national real-world cohort.

**Design, Setting, and Participants:**

This retrospective cohort study used a nationwide electronic health record–derived database of 3808 men receiving first-line therapy for mCRPC between January 1, 2012, and December 31, 2018. Data analysis was performed between January 1, 2020, and June 1, 2021. Median follow-up was 13 months (IQR, 7-22 months). Propensity score–based inverse probability of treatment weighting was applied to reduce imbalance in measured confounders between patients receiving first-line abiraterone vs other first-line therapies. Deidentified patient data originated from a geographically diverse set of approximately 280 cancer clinics (approximately 800 sites of care) throughout the United States. Participants had newly diagnosed mCRPC and were receiving first-line systemic therapy during the study period.

**Exposures:**

Receipt of abiraterone for first-line therapy.

**Main Outcomes and Measures:**

Overall survival from start of first-line treatment. Stratified analyses investigated overall survival within each race group, with first-line enzalutamide as the comparator.

**Results:**

Among 3808 patients with mCRPC, there were 2615 non-Hispanic White men (68.7%; mean [SD] age at diagnosis, 74 [8] years) and 404 African American men (10.6%; mean [SD] age at diagnosis, 69 [9] years), and 1729 patients (45.4%) in the cohort received first-line abiraterone. Among patients receiving first-line abiraterone, African American men had higher median overall survival than non-Hispanic White men (23 months [IQR, 10-37 months] vs 17 months [IQR, 9-32 months], respectively; inverse probability of treatment weighting hazard ratio, 0.76; 95% CI, 0.60-0.98). A race-by-treatment interaction existed for first-line abiraterone vs first-line enzalutamide (hazard ratio for abiraterone vs enzalutamide: non-Hispanic White men, 1.21 [95% CI, 1.06-1.38]; African American men, 1.05 [95% CI, 0.74-1.50]; interaction *P* = .02). There was no overall survival difference between first-line abiraterone and first-line enzalutamide among African American patients (24 vs 24 months, respectively; inverse probability of treatment weighting hazard ratio, 1.05; 95% CI, 0.74-1.50). First-line abiraterone was associated with decreased median overall survival relative to first-line enzalutamide among non-Hispanic White patients (17 months [IQR, 9-32 months] vs 20 months [IQR, 10-36 months], respectively; inverse probability of treatment weighting hazard ratio, 1.21; 95% CI, 1.06-1.38).

**Conclusions and Relevance:**

In this cohort study of patients who received first-line systemic therapy for mCRPC, African American men who received abiraterone had improved overall survival compared with non-Hispanic White men. Future prospective studies should assess drivers of differential abiraterone outcomes in mCRPC between African American and non-Hispanic White men, including differences in genetic factors and socioeconomic status, to inform treatment strategies.

## Key Points

**Question:**

How do outcomes associated with first-line abiraterone therapy differ by race in a real-world cohort of patients with metastatic castration-resistant prostate cancer (mCRPC)?

**Findings:**

In this propensity score–adjusted cohort study of 3808 patients with mCRPC, among men receiving first-line abiraterone, African American men had significantly longer median overall survival compared with non-Hispanic White men (23 vs 17 months). Among non-Hispanic White men, first-line abiraterone was associated with significantly lower median overall survival than enzalutamide (17 vs 20 months).

**Meaning:**

Abiraterone in mCRPC was associated with differential outcomes by race, possibly driven by decreased benefit among non-Hispanic White men.

## Teaser

This cohort study of African American and non-Hispanic White men uses the Flatiron Health database to examine survival outcomes associated with first-line abiraterone therapy for metastatic castration-resistant prostate cancer.

## Introduction

Despite multiple agents receiving approval for treatment of metastatic castration-resistant prostate cancer (mCRPC), African American men are underrepresented in seminal phase 3 trials in mCRPC.^1^ Given that African American men are more likely to develop and die from metastatic prostate cancer than non-Hispanic White men, identifying optimal treatment strategies for African American men with mCRPC is a key public health priority.^2,3^ Recent evidence suggests abiraterone is associated with improved prostate cancer outcomes for African American men compared with non-Hispanic White men.^4,5,6,7^ African American patients treated with abiraterone in the Abi-Race prospective trial had longer median time to prostate-specific antigen progression than non-Hispanic White patients (16.6 vs 11.5 months, respectively) and higher rates of at least 50% prostate-specific antigen decline (74% vs 66%, respectively); however, these differences were not statistically significant.^7^ It is unclear whether these outcomes extend to contemporary real-world cohorts, particularly given availability of other effective therapies, such as enzalutamide.

We investigated differences in outcomes associated with first-line abiraterone between African American and non-Hispanic White men with mCRPC in a national real-world cohort, which to our knowledge represents a larger sample size with greater power than that of similar investigations to date. Moreover, through comparing abiraterone with enzalutamide, our study is the first to our knowledge to evaluate for an association between race and androgen receptor signaling inhibition therapy and thereby examine both the prognostic value of race among abiraterone-treated patients and the predictive value of race on abiraterone vs enzalutamide outcomes.

## Methods

### Data Source

This retrospective cohort study used the nationwide Flatiron Health electronic health record–derived deidentified database, a longitudinal data set consisting of deidentified patient-level structured and unstructured data curated via technology-enabled abstraction. During the study period, data originated from approximately 280 cancer clinics (approximately 800 sites of care). The framework and methodology used by the Flatiron Health database to generate its mCRPC cohort is detailed extensively elsewhere.^8^ Briefly, CRPC status is identified through an algorithm incorporating physician documentation of hormone-resistant status and resistance to androgen deprivation therapy through certain prostate-specific antigen elevation parameters. Lines of therapy are documented in the database relative to the index date of mCRPC diagnosis, in which the first line of treatment was defined as the first therapeutic agent initiated after mCRPC diagnosis plus any other agents initiated within 28 days of the first agent. The University of Pennsylvania institutional review board approved the protocol with a waiver of informed consent, given use of deidentified data. This report followed the Strengthening the Reporting of Observational Studies in Epidemiology (STROBE) reporting guideline for cohort studies.

### Study Population and Covariates

The study population included patients with newly diagnosed mCRPC who were receiving first-line systemic therapy between January 1, 2012, and December 31, 2018. Patients were excluded if they had no documented first-line therapy, a greater than 90-day gap between mCRPC diagnosis and first structured electronic health record activity, or nonmetastatic CRPC; or had received first-line agents not listed in National Comprehensive Cancer Network guidelines^9^ for mCRPC (eFigure 1 in the Supplement). Follow-up was censored at death or last confirmed electronic health record activity. Race and ethnicity categorizations in the Flatiron Health database are modeled after the US Office of Management and Budget standards for race and ethnicity,^10^ which inform census categories and Food and Drug Administration guidelines for collecting race and ethnicity information in clinical trials. Race and ethnicity information was self-reported by patients and then documented by clinical teams directly within the electronic health record or within a practice management system from which data were then imported into the electronic health record.

### Statistical Analysis

Data analysis was performed between January 1, 2020, and June 1, 2021. The primary exposure was receipt of abiraterone for first-line therapy, either with androgen deprivation therapy alone or in combination with other systemic therapies. Propensity score–based inverse probability of treatment weighting (IPTW) was applied to reduce imbalance in measured confounders between patients receiving first-line abiraterone vs other first-line therapies (eFigure 2 in the Supplement). Patient-level covariates measured at mCRPC diagnosis included age, race and ethnicity, Elixhauser comorbidities,^11^ prostate-specific antigen level, docetaxel treatment in the metastatic hormone-sensitive setting, insurance type, and opioid use as a proxy of disease severity,^12^ given that prior research has demonstrated that pain is a predictor of overall survival in men with mCRPC and that opioid use correlates with higher pain scores.^13^ Practice-level covariates included treatment setting (academic vs community), geographic region, and abiraterone prescribing rate. Abiraterone prescribing rate was defined as the proportion of first-line treatment initiations containing abiraterone per practice and was used to account for any variation in baseline practice patterns^14^ that might influence patient receipt of abiraterone as part of first-line therapy. A secondary analysis assessed exposure to first-line enzalutamide treatment, using the same propensity score–based methodology and covariates.

The primary outcome was overall survival from start of first-line treatment. IPTW-adjusted Kaplan-Meier curves compared treatment-specific overall survival between African American and non-Hispanic White patients to estimate the marginal association between race and overall survival after balance was achieved in measured confounders. Cox proportional hazards regressions estimated IPTW-adjusted hazard ratios (HRs) and 95% CIs. Stratified analyses investigated overall survival within each race group, with first-line single-agent enzalutamide as the comparator. A 2-sided *P *value of .05 was considered statistically significant. All models included race-treatment interactions to determine whether differences in outcomes by race varied by treatment group. Stata/IC version 15.1 (StataCorp) was used for data analysis.

## Results

Of 3808 patients with mCRPC, 1729 (45.4%) received abiraterone-based first-line therapy, and 2079 (54.6%) received nonabiraterone-based first-line regimens (Table). The cohort included 2615 non-Hispanic White patients (68.7%; mean [SD] age, 74 [8] years) and 404 African American patients (10.6%; mean [SD] age, 69 [9] years) (eTable 1 in the Supplement). Median follow-up was 13 months (IQR, 7-22 months) for both African American and non-Hispanic White patients. For the unweighted abiraterone first-line and nonabiraterone first-line cohorts, similar proportions of patients presented with de novo metastatic disease (43% [737 of 1729] vs 44% [916 of 2079], respectively) and went on to receive second-line therapy (55% [955 of 1729] vs 56% [1160 of 2079], respectively). In the unweighted population, 1202 non-Hispanic White patients (46%) and 170 African American patients (42%) received first-line abiraterone. Among patients receiving first-line abiraterone, 94% (1630 of 1729) received single-agent abiraterone; among those receiving nonabiraterone first-line therapy, 56% (1155 of 2079) received single-agent enzalutamide.

**Table.** Baseline Characteristics of the Nonabiraterone and Abiraterone First-Line Treatment Groups

Characteristic	Unweighted population (n = 3808), No. (%)			Weighted population (n = 3064), No. (%)^a^		
	Nonabiraterone first-line treatment (n = 2079)	Abiraterone first-line treatment (n = 1729)	SMD	Nonabiraterone first-line treatment (n = 1672)	Abiraterone first-line treatment (n = 1392)	SMD
Single-agent enzalutamide, No.	1155	NA	NA	928	NA	NA
Single-agent abiraterone, No.	NA	1630	NA	NA	1312	NA
Age at CRPC diagnosis, mean (SD), y	72 (9)	74 (8)	0.16	72^b^	74^b^	0.01
Race						
African American	234 (11)	170 (10)	0.04	200 (12)	153 (11)	0.01
Non-Hispanic White	1413 (68)	1202 (70)		1254 (75)	1058 (76)	
Other^c^	252 (12)	217 (13)		234 (14)	195 (14)	
Comorbidity count, mean (SD)	3 (1)	3 (1)	0.02	2.8^b^	2.8^b^	0.01
Opioid use	162 (8)	135 (8)	0.01	134 (8)	111 (8)	0.01
PSA at metastatic diagnosis, median (IQR), ng/mL	56 (14-240)	57 (15-248)	0.03	54^b^	57^b^	0.02
Docetaxel receipt in the mHSPC setting	213 (10)	154 (9)	0.05	184 (11)	125 (9)	0.01
Practice abiraterone prescribing rate, mean (SD)	0.39 (0.12)	0.48 (0.14)	0.64	0.40^b^	0.47^b^	0.04
Region						
Midwest	252 (12)	233 (13)	0.01	217 (13)	209 (15)	0.01
Northeast	346 (17)	278 (16)		268 (16)	223 (16)	
South	977 (47)	726 (42)		819 (49)	585 (42)	
West	350 (17)	310 (18)		268 (16)	251 (18)	
PR	19 (1)	34 (2)		17 (1)	14 (1)	
Other	135 (6)	148 (9)		100 (6)	111 (8)	
Practice type						
Academic	125 (6)	141 (8)	0.08	100 (6)	111 (8)	0.01
Community	1954 (94)	1588 (92)		1572 (94)	1281 (92)	
Payer						
Commercial health plan	635 (31)	566 (33)	0.03	518 (31)	459 (33)	0.01
Public	402 (19)	306 (18)		334 (20)	251 (18)	
Other	598 (29)	485 (28)		468 (28)	376 (27)	
Not listed	444 (21)	372 (22)		351 (21)	292 (21)	

Abbreviations: CRPC, castration-resistant prostate cancer; mHSPC, metastatic hormone-sensitive prostate cancer; NA, not applicable; PR, Puerto Rico; PSA, prostate-specific antigen; SMD, standardized mean difference.

^a^ Patients with missing data for any of the covariates adjusted for in the propensity score analysis were excluded from that analysis and therefore are not represented in the weighted population.

^b^ Standard deviations or IQRs are not available for weighted population data.

^c^ Other includes American Indian or Alaska Native, Asian, Hawaiian or Pacific Islander, Hispanic or Latino, and patients for whom source data contained a race description that fell into multiple race categories.

**Figure 1.**
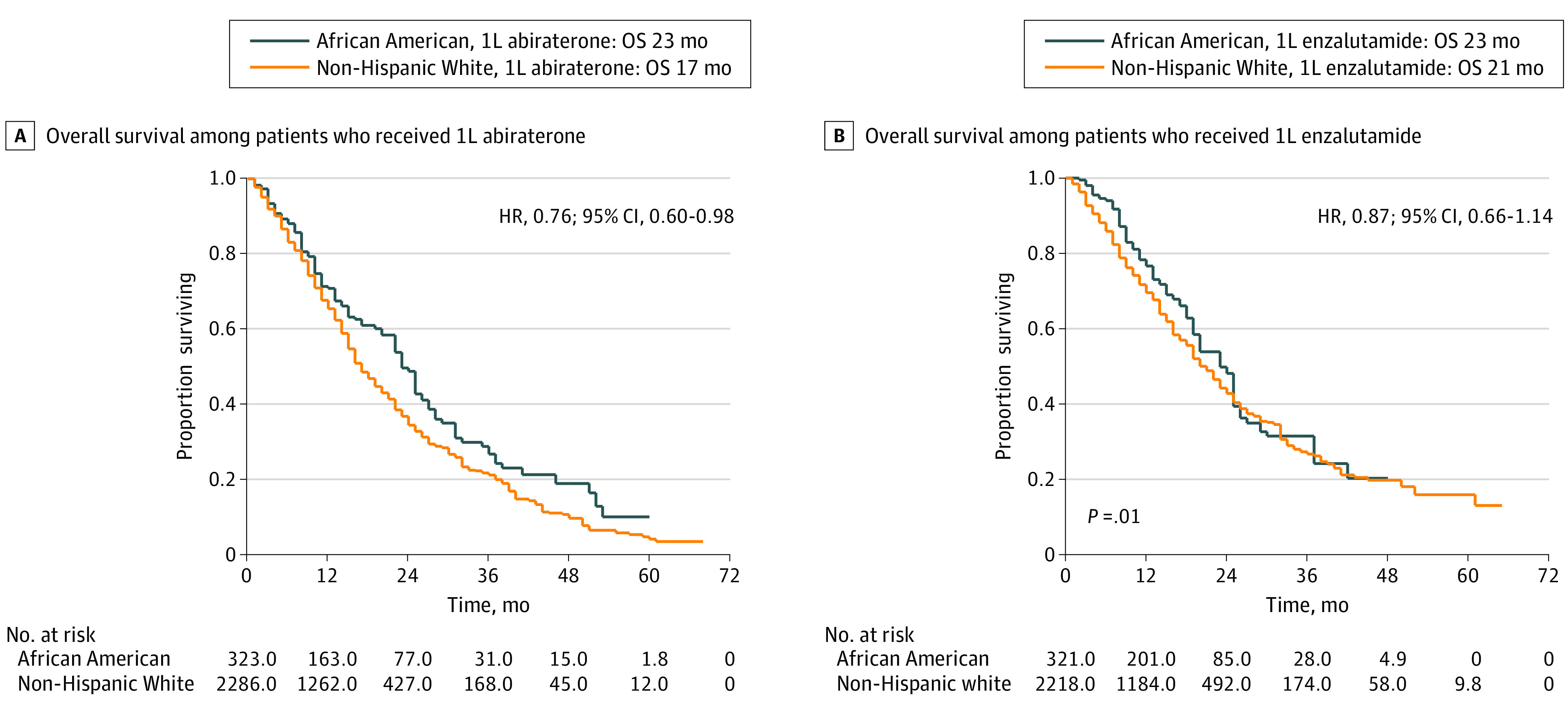
Overall Survival by Race, African American vs Non-Hispanic White Men Numbers at risk represented in Kaplan-Meier curves are scaled according to the propensity score analysis inverse probability of treatment weighting. HR indicates hazard ratio; OS, median overall survival; and 1L, first line.

**Figure 2.**
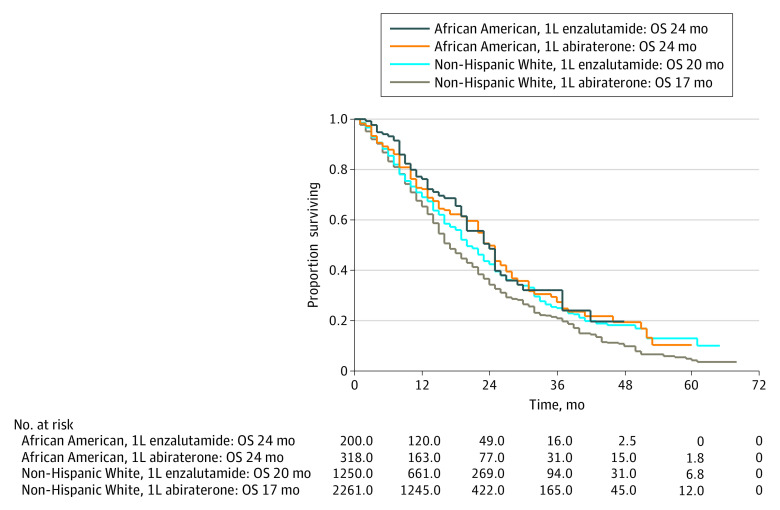
Overall Survival by Race and Treatment, First-Line Abiraterone vs First-Line Enzalutamide Treatment groups correspond to abiraterone-based first-line regimens and first-line single-agent enzalutamide. Numbers at risk represented in Kaplan-Meier curves are scaled according to the propensity score analysis inverse probability of treatment weighting. OS indicates median overall survival; 1L, first line.

Among patients receiving first-line abiraterone, median overall survival was higher among African American patients than non-Hispanic White patients (23 months [IQR, 10-37 months] vs 17 months [IQR, 9-32 months], respectively; IPTW HR for mortality, 0.76; 95% CI, 0.60-0.98) (Figure 1A). Among African American patients, first-line abiraterone was not associated with a median overall survival advantage compared with first-line single-agent enzalutamide (24 months [IQR, 11-37 months] vs 24 months [IQR, 13-37 months], respectively; IPTW HR, 1.05; 95% CI, 0.74-1.50) (Figure 2). Among non-Hispanic White patients, first-line abiraterone was associated with decreased median overall survival compared with first-line single-agent enzalutamide (17 months [IQR, 9-32 months] vs 20 months [IQR, 10-36 months], respectively; IPTW HR, 1.21; 95% CI, 1.06-1.38). Among patients receiving first-line enzalutamide-based regimens, no median overall survival difference existed between African American and non-Hispanic White patients (23 months [IQR, 13-37 months] vs 21 months [IQR, 10-38 months], respectively; IPTW HR, 0.87; 95% CI, 0.66-1.14) (Figure 1B). Significant overall survival race-by-treatment interactions existed for first-line abiraterone vs first-line nonabiraterone regimens (IPTW HR non-Hispanic White men divided by IPTW HR African American men = 1.21; HR for abiraterone vs nonabiraterone for non-Hispanic White men, 1.16 [95% CI, 1.04-1.30]; HR for abiraterone vs nonabiraterone for African American men, 0.96 [95% CI, 0.70-1.30]; interaction *P* = .03) and for first-line abiraterone vs first-line single-agent enzalutamide (IPTW HR non-Hispanic White men divided by IPTW HR African American men = 1.15; HR for abiraterone vs enzalutamide for non-Hispanic White men, 1.21 [95% CI, 1.06-1.38]; HR for abiraterone vs enzalutamide for African American men, 1.05 [95% CI, 0.74-1.50]; interaction *P* = .02) (eTables 2 and 3 in the Supplement).

## Discussion

In this real-world analysis of nearly 4000 patients with mCRPC who were receiving first-line treatment, abiraterone was associated with superior overall survival for African American men compared with non-Hispanic White men. Race-stratified analyses using enzalutamide as a comparator suggested that this overall survival disparity may have been due to decreased benefit associated with abiraterone among non-Hispanic White men. In contrast, among patients receiving first-line enzalutamide, there was no racial difference in overall survival. Favorable outcomes for African American men receiving abiraterone were previously observed in retrospective studies,^4,5^ but no significant race-based differences in abiraterone effectiveness were found in prospective data.^6,7^ It is unclear, however, whether the lack of significant differences in these prospective studies was due to insufficient power. Our study suggests that abiraterone is associated with better outcomes for African American vs non-Hispanic White men in real-world practice and extends prior analyses by investigating race-specific overall survival in a large real-world cohort. Our study demonstrated a significant race-treatment interaction association, which to our knowledge has not been reported by prior randomized studies investigating abiraterone and enzalutamide. Although there may not be a comparative advantage for abiraterone over other first-line mCRPC agents among African American men, other agents may be favored above abiraterone among non-Hispanic White men. Beyond the context of androgen receptor signaling inhibitors, there is emerging evidence for race-treatment interaction associations in the mCRPC setting with respect to sipuleucel-T therapy, for example,^15^ which likewise underscores the need to further explore the interplay between race and treatment as it relates to variation in mCRPC outcomes.

Future investigation into drivers of differential outcomes between mCRPC agents should move beyond studying race itself because race may not account for all underlying factors, including socioeconomic differences, that contribute to disparate outcomes.^16,17^ To that end, genetic variants are being explored as factors in mCRPC treatment response. The *HSD3B1*(1245A>C) gene variation has higher single-nucleotide variation allelic frequency among male individuals of European (32%) vs African American (15%) ancestry^18,19^ and has been shown to increase 3βHSD1 enzyme quantity within androgen receptor signaling pathways.^20^ Increased 3βHSD1 enzyme quantity in turn yields greater androgen production and androgen receptor activation, thereby creating a potential treatment resistance mechanism to abiraterone.^20,21,22^ If future randomized trials confirm this treatment-related association, precision oncology treatment strategies may have important applications in mCRPC and contribute to decreasing disparities in outcomes, but only in concert with systematic efforts to increase representation of non-White populations in cohort studies and clinical trials.

### Limitations

Because this cohort study was a real-world analysis, a key limitation was the inability to fully account for socioeconomic factors and biases that, in conjunction with race, contribute to receipt of a given therapy and outcomes disparities.^2,17^ Likewise, certain covariates may have been incompletely coded owing to inconsistent electronic health record documentation (eg, comorbidities), and other variables affecting prognosis and outcomes were not collected or easily analyzable in this database (eg, treatment adherence, burden of metastatic disease, family history, genetic risk factors). However, this missingness is common with observational data sets, and we have no evidence that it was unbalanced between African American and non-Hispanic White men. In addition, our analysis included a relatively short median follow-up of 13 months. Finally, non-Hispanic White men disproportionately outnumbered African American men in the study sample. However, the sample proportion of African American men exceeded typical representation of African American men in CRPC trials by more than 3-fold.^1^ Despite these limitations, to our knowledge this is the largest real-world study to date investigating differential outcomes with abiraterone use by race in the mCRPC setting.

## Conclusions

Prior research suggests that abiraterone is associated with greater clinical benefit for African American patients than for White patients with mCRPC. This real-world cohort study affirms these findings and is, to our knowledge, the first one to find that first-line abiraterone was associated with improved overall survival among African American patients compared with White patients. This observation may be due to decreased effectiveness of first-line abiraterone relative to other first-line therapies among White patients. Future prospective research in mCRPC should include greater proportions of African American men and investigate mechanisms behind potential decreased abiraterone effectiveness among White men to ultimately inform solutions to ameliorate disparities in outcomes.

## Notes

Supplement. **eTable 1.** Baseline Characteristics of African American and Non-Hispanic White Patients **eTable 2.** Race-Treatment Interaction Effects for the Abiraterone vs Nonabiraterone First-Line Treatment Regimens **eTable 3.** Race-Treatment Interaction Effects for Abiraterone vs Single-Agent Enzalutamide First-Line Treatment Regimens **eFigure 1.** CONSORT Diagram **eFigure 2.** Distribution of Propensity Scores
