# Supplementary material for: Outcomes Among African American and Non-Hispanic White Men With Metastatic Castration-Resistant Prostate Cancer With First-Line Abiraterone
Source: JAMA Netw Open. 2022 Jan 5;5(1):e2142093. doi: 10.1001/jamanetworkopen.2021.42093 (PMC8733836; doi:10.1001/jamanetworkopen.2021.42093)
Supplement: Supplement. — eTable 1. Baseline Characteristics of African American and Non-Hispanic White Patients eTable 2. Race-Treatment Interaction Effects for the Abiraterone vs Nonabiraterone First-Line Treatment Regimens eTable 3. Race-Treatment Interaction Effects for Abiraterone vs Single-Agent Enzalutamide First-Line Treatment Regimens eFigure 1. CONSORT Diagram eFigure 2. Distribution of Propensity Scores [file jamanetwopen-e2142093-s001.pdf]

Supplemental Online Content

Marar M, Long Q, Mamtani R, Narayan V, Vapiwala N, Parikh RB. Outcomes among African American and non-Hispanic White men with metastatic castration-resistant prostate cancer with first-line abiraterone. *JAMA Netw Open*. 2022;5(1):e2142093.
doi:10.1001/jamanetworkopen.2021.42093

eTable 1. Baseline Characteristics of African American and Non-Hispanic White Patients

eTable 2. Race-Treatment Interaction Effects for the Abiraterone vs Nonabiraterone First-Line Treatment Regimens

eTable 3. Race-Treatment Interaction Effects for Abiraterone vs Single-Agent Enzalutamide First-Line Treatment Regimens

eFigure 1. CONSORT Diagram

eFigure 2. Distribution of Propensity Scores

This supplemental material has been provided by the authors to give readers additional information about their work.

© 2022 Marar M et al. *JAMA Network Open.*

eTable 1. Baseline Characteristics of African American and non-Hispanic White Patients

	African-American (N=404)	White (N=2615)	p-value
	N (%)	N (%)	
**Mean age at CRPC diagnosis**	69	74	<0.001
**Mean comorbidity count**	3	3	0.28
**Opioid use**	41 (10)	224 (9)	0.30
**Median PSA at metastatic diagnosis**	126	47	<0.001
**Docetaxel receipt in the mHSPC setting**	47 (12)	253 (10)	0.22
**Practice Abiraterone prescribing rate**	.42	.43	0.15
**Region**			<0.001
Midwest	37 (9)	372 (14)	
Northeast	43 (11)	442 (17)	
Puerto Rico	4 (1)	17 (1)	
South	264 (65)	1,191 (46)	
West	30 (7)	376 (14)	
Other	26 (6)	217 (8)	
**Practice type**			0.092
Academic	23 (6)	212 (8)	
Community	381 (94)	2403 (92)	
**Payer**			0.002
Commercial Health Plan	118 (29)	850 (33)	
Other	91 (23)	747 (29)	
Public	85 (21)	492 (19)	
Not listed	110 (27)	526 (20)	

This supplemental material has been provided by the authors to give readers additional information about their work.

© 2021 Marar et al. *JAMA Network Open.*

eTable 2. Race-Treatment Interaction Effects for the Abiraterone vs Nonabiraterone First-Line Treatment Regimens

	Median overall survival in abiraterone 1L group (months)	Median overall survival in non-abiraterone 1L group (months)	Hazard ratio abiraterone 1L/ non-abiraterone 1L (95% CI)	Hazard ratio White/Hazard ratio African American
White	17	19	1.16 (1.04-1.30)	1.21<br>P* = 0.03
African American	23	23	0.96 (0.70-1.30)	

*p-value for interaction term

This supplemental material has been provided by the authors to give readers additional information about their work.

© 2021 Marar et al. *JAMA Network Open.*

eTable 3. Race-Treatment Interaction Effects for Abiraterone vs Single-Agent Enzalutamide First-Line Treatment Regimens

	Median overall survival in abiraterone 1L group (months)	Median overall survival in enzalutamide 1L group (months)	Hazard ratio abiraterone 1L/ enzalutamide 1L (95% CI)	Hazard ratio White/Hazard ratio African American
White	17	20	1.21 (1.06-1.38)	1.15<br>P* = 0.02
African American	24	24	1.05 (0.74-1.50)	

*p-value for interaction term

This supplemental material has been provided by the authors to give readers additional information about their work.

© 2021 Marar et al. *JAMA Network Open.*

eFigure 1. CONSORT Diagram

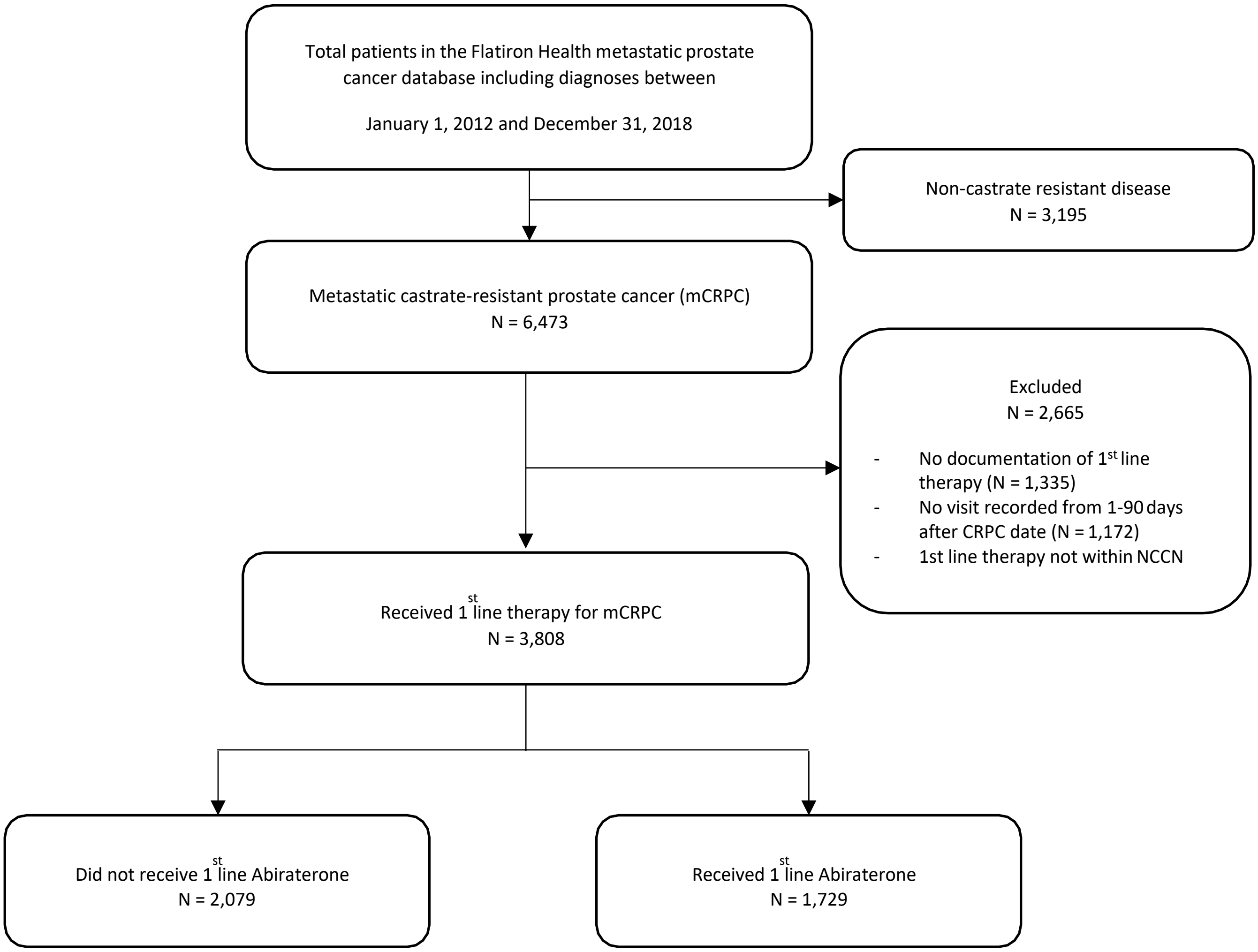

NCCN = National Comprehensive Cancer Network

This supplemental material has been provided by the authors to give readers additional information about their work.

© 2021 Marar et al. *JAMA Network Open.*

eFigure 2. Distribution of Propensity Scores

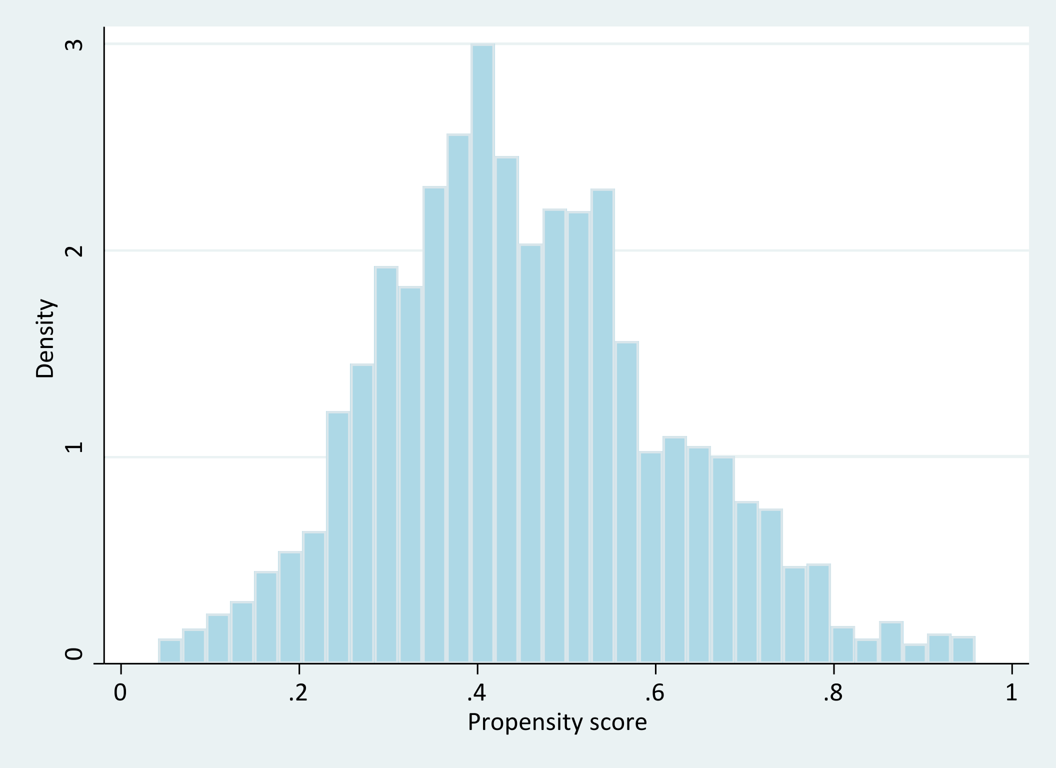

© 2021 Marar et al. *JAMA Network Open.*
